# Progress in Surgery

**Published:** 1898-01-22

**Authors:** 


### Progress in Surgery.

### TUMOURS OF THE BRAIN.

**Difficulties in Diagnosis.**—With all the progress that has been made within recent years in the surgery of brain tumours we are yet without sufficient knowledge to definitely diagnose the site, extent, and characters of the majority of these new growths. Few men have had a wider experience of cerebral tumours both from the physician's and the surgeon's point of view than Allen Starr, of New York; and when we find him writing, after a study of 220 cases in which operation has been resorted to, that "it must be affirmed that in every case an operation for the removal of a brain tumour is an exploratory operation," we need no further proof of the difficulties of diagnosis. A few cases recently reported will illustrate this:—

1. Starr and Weir,[1] while pointing out that it is a fortunate fact that the parts of the brain in which tumours can be most definitely located are the parts which are most easily accessible to the surgeon, do not forget that clinical signs are often very misleading. This is partly due to the fact that pressure is often transmitted by continuity of tissue to parts distant from the growth, and so gives rise to false symptoms. In a case recorded by them, slowly progressing motor aphasia, followed in three months by rapidly-increasing right hemiplegia and duller percussion note on the left side, led to the diagnosis of a tumour of the left third frontal convolution growing backward and inward, so as to compress the motor tract in its passage toward the internal capsule. On operating no such tumour was found, but ten days later the patient died, and an autopsy revealed a vascular infiltrating glioma deep within the apex of the temporal lobe. It was an example of pressure producing "indirect local symptoms." The case is fully reported and illustrated.

2. Barr and Nicoll,[2] of Glasgow, report a case in which the symptoms of a malignant tumour of the brain, probably originating in the middle ear, closely simulated those of temporo-sphenoidal abscess. The patient was a boy, 12½ years old, who first complained of pain, discharge from, and defective hearing in his right ear. A small mass resembling a polypus was removed, but recurrence took place, and on opening up the mastoid this cavity was found filled with the same material. Soon afterwards intra-cranial symptoms, suggestive of temporo-sphenoidal abscess, developed, and the skull was opened in the usual place, but, instead of an abscess a large tumour was found. This was removed, and the boy died two and a half months after the operation. The tumour was most probably a sarcoma, although it presented some carcinomatous features under the microscope.

3. Duncan and Maylard[3] successfully removed a large sarcoma from the brain in the case of a man aged 38, who three years previously was suddenly seized with convulsions, followed by Jacksonian symptoms suggestive of a lesion of the motor cortex. Maylard was guided in his operation by the convulsive twitchings of the left upper extremity, rather than by pain and tenderness which existed over the right parietal bone, but it was found that the motor area must have been secondarily implicated by backward pressure of the growth. For three years and four months the patient has remained well, but for one or two slight fits probably attributable to cicatricial adhesions.

4. Hitzig[4] reports four cases illustrative of the difficulties of diagnosis, and of want of success from operation. One of these was diagnosed a cortical tumour, the symptoms being weakness and convulsive movements in the right arm, followed later by weakness of the right leg and headache. There was no optic neuritis or sickness. On exploring the brain, no such tumour was found, and later optic neuritis, sickness, convulsive seizures of both legs, and unconsciousness followed, and the patient died. A large sarcoma, apparently commencing in the centrum ovale and spreading into the cortex and corpus callosum, was found post-mortem. In another patient, a woman 23 years of age, an operation failed to confirm the diagnosis of cerebellar tumour, which was based on the presence of vomiting, giddiness, gradual failure of vision in both eyes, with internal strabismus first of left and later of right eye. There were also present optic neuritis, slight spastic paralysis of the left extremities, and weakness of the right side of the face. On post-mortem examination, a large cystic glioma of the frontal lobe was found, flattening out the optic, third, fifth, and sixth nerves, and compressing the right half of the pons. In a third patient, operation failed alike to discover a tumour and to relieve the patient, who suffered from convulsive attacks and weakness of the right arm, beginning in the fingers and slightly affecting the head and leg, without loss of consciousness, but with laboured speech. In the fourth case a tumour was localised, found, and removed, but the patient died of shock.

There is often much difficulty in distinguishing between tumours of the prefrontal region and those of the cerebellum. In a recent number of the *American Medico-Surgical Bulletin* a case in point is refrered to. The skull was opened in the temporal region, but on post-mortem examination the tumour was found to be cerebellar. An examination of the symptoms present will show the difficulty which existed in this case. They were marked right-sided frontal pain, failure of vision beginning in the right eye, complete loss of smell, also beginning on the right side. Absence of typical cerebellar signs, such as locomotory disturbances, swaying of the body, or paralysis. There was slight exaggeration of the knee reflexes. There was no mental aberration. The following case, reported by Collins and Brewer of New York,[5] is of interest because, although the diagnosis was accurately made, several confusing symptoms were present. The patient was a man, aged 26, in whom a sudden onset of pain in the back of the head marked the beginning of his illness. This was followed by uncertainty of gait, so that "he could not keep out of the gutter" on going along the street. Vomiting and failure of sight ensued about a month later, and the pain became frontal sometimes. Weakness and emaciation were marked, but special senses and muscular functions were normal. Beyond a disinclination for conversation there was no psychical disturbance. A slight degree of choked disc developed, and the patient became sleepless. Within a few months he lost about 50 lbs. in weight. In the absence of more definite localising signs the diagnosis remained uncertain, but an exploratory operation in the cerebellar fossa was recommended and performed, and a tubercular tumour was removed from the region of the roof of the fourth ventricle on the right side. Two months later he remained free from pain and vomiting, his eyesight had almost become normal, and he had gained 30 lbs. in weight. Two specially interesting facts were observed during the convalescence, namely, that he complained of a continuous salty taste in the mouth for three or four weeks, and there was marked polyuria. In discussing the merits of operation for cerebellar tumours the authors are inclined to urge further trial of it, although results published have been far from encouraging. One of the most successful reported was that operated on by Annandale, of Edinburgh, where the tumour was a sarcoma. In the case under discussion, that of Collins and Brewer, the improvement was not maintained, the patient dying from recurrence in a few months. Post mortem the growth was found to be a caseous tuberculoma.

**Some Points in the Operative Procedure in Cases of Cerebral Tumours.**—Keen[6] discusses some points in technique. He mentions the various methods by which the skull may be opened. Doyen, who ignores localising symptoms, removes a very large portion of one side of the skull, and exposing a corresponding area of the brain, searches for the tumour. His results are not encouraging. The trephine is still the favourite instrument, at least with British surgeons. Horsley uses a straight saw, Krause a circular saw driven by a dental engine, Hartley and Pyle prefer chisels, and Cryer uses drills. Keen advises with some other American surgeons, that the operation should be done in two stages, a few weeks intervening. Shock and hæmorrhage are thus diminished. Hæmorrhage is met by ordinary surgical means. The tumour may be removed by the fingers, knife, or spoon, and even when removal is incomplete much relief is afforded the patient. When much dura mater is removed Keen advises that the pericranium, turned inside out, be utilised to replace it. Drainage on the whole is recommended. The opening in the skull may be closed by replacing the bone in pieces (Macewen) or in bulk. Konig has used the outer and part of the middle table from the adjacent area to cover in large gaps. Foreign bodies like celluloid, silver and gold plates have been employed with satisfactory results.

**Chloroform in Cerebellar Operations.**—It is a common experience that during operations on the cerebellum, patients sometimes give trouble apparently associated with the anæsthetic. Macewen refers to cases in his work on "Pyogenic Diseases of the Brain and Spinal Cord," and other surgeons have recorded similar cases. Barr and Nicoll, in their operation above referred to, met with this difficulty. Within ten minutes of the beginning of the operation tracheotomy and artificial respiration had to be resorted to for arrest of breathing, the heart meanwhile continuing to act well. The skull was opened while the artificial respiration was being carried on, and the relief of tension following this enabled the voluntary respiration to proceed. Consciousness was speedily regained. Clark had a similar experience in a case of cerebellar abscess. Tracheotomy was performed, and artificial respiration kept up for twenty-four hours. The patient died. Collins and Brewer[5] had trouble of the same kind in their case already quoted. Ether was the anæsthetic used, and just as the cerebellum was exposed the patient was observed to be pulseless. Whisky and hot water enemata were administered, and salt solution injected into the veins of the arm, and the patient rallied. Later in the operation the pulse again gave cause for anxiety, and saline solution was again injected with good results. In this case the respiration apparently went on.

[1] Med. News, Aug. 7, 1897. [2] Glasgow Med. Jour., Sep. 1897. [3] Glasgow Med. Jour., April, 1897. [4] Lancet (abstract), March 19, 1897. [5] Med. Record, May 15, 1897. [6] University Med. Mag., March, 1896.

